# Mental Health in Ethnic Minority Populations in the UK: Developmental Trajectories from Early Childhood to Mid Adolescence

**DOI:** 10.1007/s10964-021-01481-5

**Published:** 2021-08-26

**Authors:** Simran Bains, Leslie Morrison Gutman

**Affiliations:** University College London, 1-19 Torrington Place, London, WC1E 7HB UK

**Keywords:** Ethnic differences, Child mental health, Developmental trajectories, UK Millennium Cohort Study, Internalizing problems, Externalizing problems

## Abstract

A large body of literature has demonstrated that there are developmental differences in mental health problems. However, less is known about the development of mental health problems in ethnic minority children, particularly at the population level. Using a detailed ethnic classification and nationally representative data from the UK Millennium Cohort Study (*n* = 18, 521, 49% female, 18% ethnic minority), this study examines ethnic differences in children’s mental health problems and trajectories of mental health from ages 3 to 14 years. Growth curve modeling revealed that ethnic minority children followed different developmental trajectories of internalizing and externalizing problems than white children, either in terms of the mean-level and/or rate of change across age. These differences were not explained by child sex, socioeconomic status, maternal depressive symptoms, and maternal immigrant status, highlighting the need for further research exploring the factors that underpin ethnic inequalities in child mental health.

## Introduction

Mental health problems affect 1 in 8 children aged 5 to 19 in the UK (Sadler et al., 2018), with 50% of clinically diagnosable disorders developing by age 14 (World Health Organization, 2018). They are characterized by internalizing (anxiety disorder, major depressive disorder) and externalizing (conduct disorder, attention-deficit/hyperactivity disorder) problems. A large body of literature has demonstrated that there are developmental differences in mental health problems (see Vella et al., 2019, for a recent review). Such research maps developmental trajectories of mental health, shedding light on the typical age of onset and relative risk throughout childhood. However, few studies have assessed the developmental course of mental health among ethnic minority children in the UK. This research gap is important to fill, given that such explorations can identify mental health inequalities, influence the content and timing of primary prevention programs, and help tailor culturally sensitive clinical interventions. Using data from the UK Millennium Cohort Study (MCS), a nationally representative and ethnically diverse longitudinal survey, this study aims to fill this gap by investigating ethnic differences in developmental trajectories of internalizing and externalizing problems from early childhood to mid adolescence.

### Ethnic Differences in Mental Health Problems

Existing evidence shows that there are ethnic differences in the diagnosis and prevalence of children’s mental health problems. In the US, African American, Asian American, and Hispanic youth are more likely to be diagnosed with disruptive behavior disorder and conduct-related problems than non-Hispanic, white youth (Mak & Rosenblatt, 2002; Nguyen et al., 2007), while higher levels of depressive symptomology have been consistently found among Hispanic adolescents (McLaughlin et al., 2007). The US and UK differ, however, with respect to ethnic composition. In the US, around 40% of the population are from African American (13%), Hispanic (18%) and other minority backgrounds (U.S. Department of Commerce, 2019), while in the UK, only 14% of the population identify as Black African (1.8%), Black Caribbean (1.1%), Indian (2.5%), Pakistani (2%), Bangladeshi (.8%), mixed (2.2%) or other (3.6%) minority ethnic (Office for National Statistics, 2011a). Given that the ethnic classifications and ethnic densities differ between the two countries, the findings from US-based research are likely to be contextually specific and not applicable to the UK.

Ethnic differences in child mental health have also been found in the UK context. Using data from the MCS, one study examined composite mental health scores among 3-year-olds, finding that Indian and Pakistani/Bangladeshi (aggregated group) children had significantly more mental health problems than white children, while Black African children had fewer problems (Platt, 2012). Another study assessed mental health scores among 7-year-olds, also using data from the MCS. They found that Pakistani, Bangladeshi, and Black Caribbean children experienced significantly more internalizing problems than white children. Pakistani and Black Caribbean children displayed more, while Black African children displayed fewer, externalizing problems than white children (Zilanawala et al., 2015). Overall, these findings suggest that ethnic minority children (with the exception of Black Africans) may experience worse mental health than white children. Rather than taking a developmental perspective, however, these studies focus on ethnic differences in mental health in only one age group. It is therefore unclear if there are age-related trends in ethnic minority mental health in the UK.

In the UK, ethnic differences in adolescent mental health have also been documented. Using data from the Research with East London Adolescents: Community Health Survey (RELACHS), two studies assessed mental health among 11- to 14-year-olds, finding that Black and South Asian adolescents had significantly fewer mental health problems than white adolescents (Fagg et al., 2006; Stansfeld et al., 2004). In both studies, mental health was operationalized using the total difficulties score from the Strengths and Difficulties Questionnaire (SDQ; Goodman, 1997). Other studies have measured the SDQ total difficulties score among 11- to 16-year-olds, using data from the Determinants of Adolescent Social wellbeing and Health (DASH) study. They found that ethnic minority adolescents reported better mental health than white adolescents (Astell-Burt et al., 2012; Harding et al., 2015), despite experiencing more adversity in terms of economic deprivation and racial discrimination. This advantage persisted after adjusting for parenting style (Maynard & Harding, 2010a) and time spent in family activities (Maynard & Harding, 2010b). Taken together, these studies suggest that ethnic minority adolescents may enjoy better mental health than their white counterparts. These findings contrast those reported in childhood (Platt, 2012; Zilanawala et al., 2015), suggesting possible developmental differences. It should be noted, however, that these studies examine data from convenience samples; thus, it is unclear whether these trends will be found using a population-based sample.

### Ethnic Differences in Developmental Trajectories of Mental Health

Longitudinal research has documented developmental differences in mental health problems (i.e., Parkes et al., 2016; Vella et al., 2019). Such research maps developmental trajectories of mental health problems, shedding light on the typical age of onset and relative risk throughout childhood. The conventional approach uses general linear or growth curve modeling, which estimate changes in mental health symptoms in a single population, across time (Jung & Wickrama, 2008). The aim of this approach is to illustrate the average trajectory of mental health for a specified population, in terms of the mean level of symptoms and course of age-related changes. These explorations have informed our understanding of mental health by showing that, for the average child, internalizing problems increase as they enter adolescence (Costello et al., 2011), and then decrease as they move towards early adulthood (Petersen et al., 2018). Externalizing problems, on the other hand, generally decline in frequency between early childhood and adolescence (Pingault et al., 2015; Miner & Clarke-Stewart, 2008).

A number of studies elucidate developmental differences in mental health problems according to individual characteristics such as sex, socioeconomic status (SES) and ethnicity. Females, for instance, show more internalizing problems than males, with differences emerging or widening following puberty (Angold et al., 1998). Males, on the other hand, display more externalizing problems than females at all ages (Lahey et al., 2000). The influence of SES on child mental health is well established, with research suggesting that children from low-income backgrounds are twice as likely to have depression than those from high income backgrounds (Gilman et al., 2002), and children living in poverty and deprivation are at significantly greater risk of conduct problems at all ages (Gutman et al., 2019).

The longitudinal relationship between ethnicity and mental health, which is the focus of this study, has received less attention. In the US, a few studies have examined ethnic differences in developmental trajectories of internalizing and externalizing problems. Using growth curve modeling, one monograph investigated the trajectories of a predominately middle-class, community-based sample of African American and European American adolescents from ages 12 to 20, finding no ethnic differences in the mean-level and slopes of adolescent-reported depressive symptoms and delinquent behaviors, where *p* < 0.01 (Gutman et al., 2017a; 2017b). Using a person-centered approach, another study assessed internalizing and externalizing trajectories from ages 6 to 18, finding that African American children were more likely to be in the chronic co-occurring, moderate co-occurring, and pure-externalizing subtype than non-Hispanic white children (Shi et al., 2020).

To date, only two studies have examined developmental trajectories of mental health among ethnic minority children in the UK. Using data from the MCS, Gutman (2019) examined ethnic differences in group-based trajectories of conduct problems, according to broad categories of ethnicity. They found that from ages 3 to 14, Black, Asian, and ‘mixed’ children followed significantly different trajectories of conduct problems than white children, both in terms of the age of onset and developmental course. The shape and pattern of conduct problem pathways differed among the three ethnic groups, respectively. Another study explored growth curve trajectories of mental health problems from ages 3 to 11, focusing specifically on mixed ethnicity in comparison to non-mixed ethnicity children. Using MCS data, they found that Pakistani mixed and Bangladeshi mixed children were at a greater risk of mental health problems in adolescence than their non-mixed peers (Zilanawala et al., 2018).

While both studies suggest that ethnic minority children may follow different developmental trajectories of mental health problems than white children, there is no research to date that has examined ethnic differences in internalizing and externalizing trajectories for specific ethnic groups in the UK from early childhood to adolescence. When collecting UK data, a harmonized approach is recommended by the Office for National Statistics (ONS) to allow consistency and comparability of statistical outputs across GB and the UK (ONS, 2015). ONS offers broader categories as well as a more specific categorization of ethnic groups. For example, the broader category ‘Asian’ comprises Indian, Pakistani, and Bangladeshi individuals, while ‘Black’ comprises Black African and Black Caribbean individuals. It is well established that using broader categories of ethnicity (such as ‘Black’ and ‘Asian’) may mask potential intra-ethnic differences within these groups (Platt, 2011; 2020). This is because intra-ethnic identities in the UK differ in terms of cultural practices, religious beliefs, socioeconomic profiles, and migration patterns to the UK (Nazroo et al., 2004). Black Caribbean and Indian migration to the UK, for instance, occurred in the 1950s and 1960s, Pakistanis in the 1960s and 1970s, Bangladeshis in the 1980s, and Black Africans in the 1990s (Kelly et al., 2009). In terms of religious differences, British Indians are generally Hindu or Sikh, while British Pakistanis and Bangladeshis are mostly Muslim, with the latter experiencing higher levels of chronic stress and racial discrimination compared with other South Asian religious groups in the UK (Williams et al., 2010). Indians in the UK have also achieved similar occupational status to white people, while Pakistani, Bangladeshi, and Black Caribbean individuals remain underrepresented in higher occupational classes (Office for National Statistics, 2011b). Given broader social and institutional nuances, these ethnic groups should be treated as heterogeneous where possible. Understanding the distinct trajectories of mental health among specific ethnic minority groups in the UK is crucial, given that it has implications for early intervention, resource use, and treatment planning.

## Current Study

Drawing upon the UK MCS, a nationally representative sample of children born around the new millennium, this study aims to elucidate the development of internalizing and externalizing problems for ethnic minority children in the UK. Based on ONS guidance, the study examines specific ethnic categories in the UK including Indian, Pakistani, Bangladeshi, Black African and Black Caribbean, given the cultural and historical heterogeneity of such groups. First, this study examines ethnic variation in children’s internalizing and externalizing problems at different ages. Based on past research, it is expected that ethnic minority children will have similar or more internalizing and externalizing problems than white children, while ethnic minority adolescents will have similar or fewer problems than white adolescents. Then, this study investigates ethnic variation in trajectories of internalizing and externalizing problems from early childhood to mid adolescence. Using growth curve modeling, developmental trajectories of internalizing and externalizing problems from ages 3 to 14 are estimated. Ethnic differences are examined, both in terms of the mean-level (i.e., intercept) and rates of change (i.e., linear and quadratic slopes) across age. Child’s sex, household income, parental education, maternal depressive symptoms, and maternal immigrant status are included as covariates to determine whether there are ethnic differences when these potentially confounding factors are accounted for. Given that past research on ethnic differences in child mental health is, for the most part, not nationally representative and sparse in the UK context, no firm hypotheses are offered. However, it is expected that developmental differences among ethnic groups will emerge.

## Methods

### Sample

The UK Millennium Cohort Study (MCS) is a population based, multidisciplinary, and prospective cohort study. It follows the development of around 18,000 children born in the UK between September 2000 and January 2002. The first survey was conducted when cohort members were 9 months old, and five follow-up surveys took place at ages 3, 5, 7, 11, and 14. A further data collection took place at age 17, but this dataset was not available on the public domain at the time of this study. To ensure adequate representation of the UK population, the MCS uses a geographically clustered and disproportionately stratified sample design. The survey oversampled smaller subpopulations such as children of ethnic minority background, children of disadvantaged background, and children living in Northern Ireland, Scotland, and Wales. This design ensures that hard to reach communities are represented and that sample sizes are adequate for the analysis of these groups. As there was an unequal probability of being sampled, sample weights are provided by the MCS survey team to account for disproportionate stratification (Plewis, 2010).

Data were gathered at each age through interviews and self-completed questionnaires. During interviews, parents were asked about their child’s ethnic background, mental health, and socioeconomic circumstances. The main informant was primarily the child’s birth mother (99% at MCS1, 96% at MCS6) and informed consent was obtained from parents prior to interviews. Ethical approval for the MCS was commissioned by the NHS Multi-Centre Research Ethics Committee (MREC). As this study is covered by the provisions of the original ethical approval (Connelly & Platt, 2014), no further ethical clearance was needed.

The number of families who have been interviewed at least once is 19,243, including 692 families in England who were not recruited until MCS2. If these cases are counted, the initial response rate was 71%. In this study, the sample included one child per family, excluding children who were the second or third in sets of twins and triplets. Growth curve modeling was based on 16,810 children for internalizing problems and 16,791 children for externalizing problems. The predictors of non-response include younger and less educated parents, not breastfeeding, renting the home, moving home, and various forms of partial response to previous surveys (Mostafa, 2014). In terms of ethnicity, Black African parents were shown to be less likely, while Pakistani/Bangladeshi parents were more likely, to respond compared to white parents. There were no other ethnic differences in the response rate. The MCS survey team has developed attrition weights to correct for biases due to non-response, alongside sample weights which account for the complex sample design (Hansen, 2014).

### Ethnicity

Ethnicity was measured at MCS1 and MCS2, when children were 9 months and 3 years of age, through a self-completed questionnaire. Parents were asked to identify the option most suitable to their child from a list of 18 ethnic identities, similar to those provided in the UK Census (ONS, 2009). Based on ONS guidance, this study collapsed responses into seven ethnic categories: White, Indian, Pakistani, Bangladeshi, Black Caribbean (including mixed White and Black Caribbean), Black African (including mixed White and Black African), and other/mixed. The ‘other/mixed’ category consists of ethnic minority children that could not be placed into one of the otherwise defined groups. The ethnic composition of the sample is shown in Table 1.

**Table 1:** Achieved sample (families) at MCS1 by ethnicity

Ethnicity	Frequency	Percentage
White	15,212	82.1
Indian	522	2.8
Pakistani	931	5.0
Bangladeshi	379	2.0
Black Caribbean	496	2.7
Black African	466	2.5
Other/mixed	515	2.8
Total	18,521	100.0

### Internalizing and Externalizing Problems

Internalizing and externalizing problems were measured at ages 3, 5, 7, 11, and 14, using the parent-reported version of the SDQ. The SDQ is a 25-item screening tool which assesses emotional and behavioral problems in the past 6 months. It has been validated for the study of children (Goodman & Goodman, 2011) and ethnic minority populations (Meltzer et al., 2003). The questionnaire consists of four problem scales (emotional symptoms, peer problems, hyperactivity/inattention, and conduct problems) and a prosocial behavior scale. The scales have five items each and each item is ranked on a three-point scale (0 = not true; 2 = certainly true). The use of the broader internalizing and externalizing SDQ scales is recommended for analyses in low-risk, population-based samples (Goodman et al., 2010).

#### Internalizing problems

In line with recommended practice, internalizing problems was calculated by summing the 10 items from the emotional symptoms and peer problems scales. Previous research has found that the internalizing problems SDQ scale showed good convergent and discriminant validity across informants (including parents) and with respect to clinical disorder (Goodman et al., 2010). Internalizing problems range from 0 to 20, with higher scores indicating more problems. In the MCS, internal reliability has been ascertained for the internalizing problems SDQ scale at ages 3, 5, 7, 11, and 14. Internal reliability was adequate, ranging from α = 0.61 (age 3) to α = 0.77 (ages 11 and 14) (Flouri et al., 2019).

#### Externalizing problems

In line with recommended practice, externalizing problems was calculated by summing the 10 items from the hyperactivity/inattention and conduct problems scales. Previous research has found that the externalizing problems SDQ scale showed good convergent and discriminant validity across informants (including parents) and with respect to clinical disorder (Goodman et al., 2010). Externalizing problems range from 0 to 20, with higher scores indicating more problems. In the MCS, internal reliability has been ascertained for the externalizing problems SDQ scale at ages 3, 5, 7, 11, and 14. Internal reliability was acceptable, ranging from α = 0.78 (ages 3 and 5) to α = 0.81 (ages 11 and 14) (Flouri et al., 2019).

### Covariates

Potential covariates were selected a priori based on existing evidence. Child level covariates included sex, while family level covariates included household income, parental education, maternal depressive symptoms, and maternal immigrant status. All covariates were measured at MCS1, when the child was 9 months of age.

#### Child sex

Girls in the UK are more likely to develop internalizing problems (Green et al., 2005) while boys are more likely to develop externalizing problems (Gutman et al. 2018), thus this study controlled for child sex. Sex was coded as a binary variable (0 = male; 1 = female).

#### Household income

Ethnic minority children in the UK are more likely to live in households facing social inequalities such as low income (Department for Work and Pensions, 2018). As early socioeconomic disadvantage is a risk factor for severe and long-term mental health problems (World Health Organization, 2014), this study controlled for household income. A measure of net household income is provided by the MCS survey team, where families are divided into five equivalized income bands (1 = lowest quantile; 5 = highest quantile).

#### Parental education

First and second-generation ethnic minority immigrants in the UK often have fewer academic qualifications than their white native counterparts (Dex & Joshi, 2004). As low parental education is associated with higher rates of mental illness in offspring (Reiss, 2013), this study controlled for parental education. Education status was defined as the mother’s highest academic qualification at the time of interview. Families were divided into six bands (0 = no formal qualifications from school or college; 5 = professional degree).

#### Maternal depressive symptoms (alpha = 0.72)

Ethnic minority mothers in the UK experience higher rates of psychiatric disorder than white mothers (Watson et al., 2019). As poor maternal mental health is predictive of children’s conduct (Gutman et al., 2019) and internalizing problems (Gutman & McMaster, 2020), this study controlled for maternal depressive symptoms. Mothers answered 9 items from a modified version of the Rutter Malaise Inventory (Rutter et al., 1970) including, “Do you often feel miserable or depressed?” and “Do you feel tired most of the time?” (0 = no; 1 = yes).

#### Maternal immigrant status

Immigrant mothers in the UK experience higher levels of chronic stress and discrimination (Fernández-Reino, 2020) and are at greater risk of developing mental health problems (Moore et al., 2019) than native-born mothers. As poor maternal mental health is predictive of children’s mental health problems, this study controlled for maternal immigrant status. Immigrant status was defined as the mother’s birthplace (0 = foreign-born mother; 1 = UK-born mother).

### Statistical Analysis

The SPSS *Complex Samples* command was used in the following analyses, in order to account for the clustered and stratified sample design of the MCS. Attrition weights were applied throughout to correct for the presence of selective attrition. For descriptive results, the mean scores and standard errors of the outcome variables were computed at each age by ethnic group. Post-hoc t-tests then assessed the statistical significance of any group differences, after Bonferroni adjustment to account for the 6 tests. White was the most prevalent ethnic category and was used as the reference category in all pairwise comparisons.

For growth curve modeling, two-level models, examining the relationship between ethnic group (level 2) and mental health problems over age (level 1), were fitted. Growth curve modeling allows for the estimation of individual mental health problem trajectories by specifying an independent variable for age (measured in child’s age in years). This type of analysis also makes it possible to elucidate variations in mental health over age, across ethnicities. Models were examined using the maximum likelihood estimation (MLE), which allows for the estimation of models with partially missing data. Modeling was completed sequentially using a series of nested models. The sequence of models fitted was as follows. Model 1 was unconditional and explored the mean-levels of internalizing and externalizing problems. Model 2 added age (linear slope for age) and Model 3 added age^2^ (quadratic slope for age). Model 4 added the child level and family level covariates. Model 5 added the interaction between age and ethnicity to assess ethnic differences in the linear slope and Model 6 added the interaction between age^2^ and ethnicity to assess ethnic differences in the quadratic slope. All statistical analysis was conducted using *SPSS v26*.

## Results

### Ethnic Differences in Mental Health Problems

Table 2 displays mean scores, standard errors, and sample sizes at each age according to ethnicity. Post-hoc t-tests identified differences between ethnic minority children and white children. All significant pairwise comparisons are illustrated using superscripts.

**Table 2:** Strengths and difficulties questionnaire (SDQ) scores by ethnicity

	White		Indian		Pakistani		Bangladeshi		Black Caribbean		Black African		Other/mixed	
	Mean (SE)	*N*	Mean (SE)	*N*	Mean (SE)	*N*	Mean (SE)	*N*	Mean (SE)	*N*	Mean (SE)	*N*	Mean (SE)	*N*
Internalizing score														
Age 3	2.74 (0.03)	11,875	3.25 (0.22)	345	4.86 (0.16)*	506	4.46 (0.30)*	171	3.20 (0.17)*	325	3.23 (0.24)	261	3.35 (0.18)*	306
Age 5	2.36 (0.03)	11,902	2.83 (0.20)	365	4.12 (0.17)*	549	3.45 (0.27)*	190	2.91 (0.20)*	324	2.98 (0.22)*	282	3.12 (0.17)*	325
Age 7	2.68 (0.04)	10,866	3.00 (0.25)	329	4.11 (0.15)*	533	4.16 (0.23)*	190	3.50 (0.18)*	286	3.06 (0.22)	262	3.13 (0.23)	286
Age 11	3.31 (0.05)	10,256	3.02 (0.21)	305	4.09 (0.16)*	551	3.97 (0.19)*	231	3.82 (0.21)	270	3.21 (0.27)	259	3.77 (0.23)	292
Age 14	3.95 (0.06)	8897	4.00 (0.70)	312	4.76 (0.18)*	568	4.43 (0.27)	243	4.61 (0.26)	228	3.64 (0.23)	234	4.10 (0.24)	284
Externalizing score														
Age 3	6.67 (0.06)	11,899	6.52 (0.29)	350	7.92 (0.20)*	491	7.25 (0.31)	171	7.47 (0.25)*	328	6.12 (0.24)	258	6.71 (0.25)	301
Age 5	4.74 (0.05)	11,907	4.54 (0.21)	361	5.64 (0.18)*	537	4.98 (0.32)	186	5.73 (0.18)*	321	4.76 (0.29)	280	4.68 (0.22)	318
Age 7	4.83 (0.05)	10,873	4.67 (0.26)	331	5.67 (0.16)*	528	4.81 (0.21)	186	5.77 (0.23)*	285	4.18 (0.26)	261	4.25 (0.28)	289
Age 11	4.75 (0.06)	10,255	4.23 (0.29)	302	4.92 (0.11)	543	4.29 (0.22)	227	5.51 (0.26)*	270	4.28 (0.33)	257	4.55 (0.26)	288
Age 14	4.81 (0.07)	8897	4.42 (0.57)	309	4.98 (0.22)	566	4.32 (0.23)	241	5.42 (0.28)	229	4.32 (0.25)	232	4.66 (0.27)	284

Post-hoc analyses using Bonferroni’s method identified significant pairwise comparisons. All two-sample t-tests use the White mean score as the reference value. Ns are unweighted, means and standard errors are weighted

*p < 0.0083

Five of the six ethnic minority groups displayed more internalizing problems than the white group. Pakistani children had significantly more internalizing problems than white children at ages 3, 5, 7, 11, and 14. Bangladeshi children had significantly more internalizing problems than white children at ages 3, 5, 7, and 11. Black Caribbean children had significantly more internalizing problems than white children at ages 3, 5, and 7. Other/mixed children had significantly more internalizing problems than white children at ages 3 and 5. Black African children had significantly more internalizing problems than white children at age 5.

Two of the six ethnic minority groups displayed more externalizing problems than the white group. Black Caribbean children had significantly more externalizing problems than white children at ages 3, 5, 7, and 11. Pakistani children had significantly more externalizing problems than white children at ages 3, 5, and 7.

### Ethnic Differences in Developmental Trajectories of Mental Health

Growth curve models were plotted controlling for child sex, household income, parental education, maternal depressive symptoms, and maternal immigrant status. For both internalizing and externalizing problems, the quadratic interactions among ethnicity were not significant, and were excluded from the models for the sake of parsimony. Model 5 is reported.

#### Internalizing problems

Regression coefficients are displayed in Table 3. The intercept shows that Indian, Pakistani, Bangladeshi, Black Caribbean, Black African, and other/mixed children had significantly higher levels of internalizing problems than white children at age 3. Boys, children whose families had lower household income, children whose parents had lower academic qualifications, and children whose mothers had more depressive symptoms also had higher levels of internalizing problems. On average, children experienced a linear decrease and slight quadratic increase in internalizing problems across age. There was a greater rate of linear decrease for Indian, Pakistani, Bangladeshi, Black African, and other/mixed children than for white children.

**Table 3:** Growth curve model for internalizing problems

Parameter	Coefficient	Standard error
Model 5		
Intercept	4.06***	0.093
Age (linear slope)	−0.20***	0.012
Age squared (quadratic slope)	0.017***	0.0007
Indian	0.96***^a^	0.15
Pakistani	1.57***^a^	0.13
Bangladeshi	1.42***^a^	0.23
Black Caribbean	0.31*^a^	0.15
Black African	0.73***^a^	0.19
Other/Mixed	0.85***^a^	0.17
Child sex	−0.13***	0.033
Household income	−0.22***	0.014
Parental education	−0.20***	0.014
Maternal mental health	0.29***	0.0096
Maternal immigrant status	−0.12	0.067
Indian * Age	−0.12***^a^	0.019
Pakistani * Age	−0.12***^a^	0.015
Bangladeshi * Age	−0.11***^a^	0.026
Black Caribbean * Age	−0.0089	0.020
Black African * Age	−0.11***^a^	0.023
Other/mixed * Age	−0.056**^a^	0.020

*p < 0.05, **p < 0.01, ***p < 0.001

^a^Significant difference between respective group and white group

**Fig. 1:**
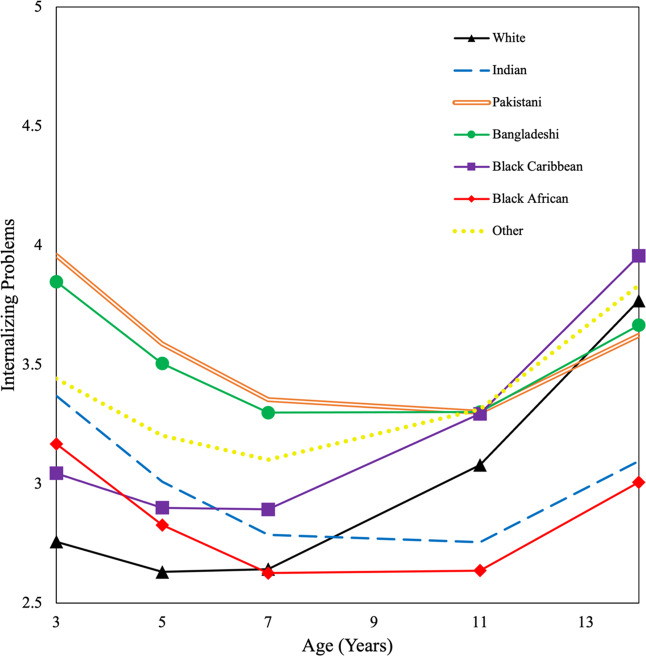
Growth curves for internalizing problems by ethnicity

Figure 1 shows the trajectories of internalizing problems according to ethnicity. From ages 3 to 7, ethnic minority children showed higher levels of internalizing problems than white children, except for Black African children who showed similar levels at age 7. Pakistani and Bangladeshi children showed the highest problem levels of all groups in early childhood. From ages 11 to 14, Pakistani, Bangladeshi, Black Caribbean, and other/mixed adolescents showed higher or similar, while Indian and Black African adolescents showed lower, levels of internalizing problems than white adolescents. There seemed to be an increase in internalizing problems from age 7 for white, Black Caribbean, and other/mixed children, and from age 11 for Indian, Pakistani, Bangladeshi, and Black African children. By age 14, Black Caribbean adolescents showed the highest problem levels of all groups, while Black African adolescents showed the lowest.

#### Externalizing problems

Regression coefficients are displayed in Table 4. The intercept shows that Pakistani and Black Caribbean children had significantly higher levels of externalizing problems than white children at age 3. Boys, children whose families had lower household income, children whose parents had lower educational qualifications, children whose mothers had more depressive symptoms, and children whose mothers were born in the UK also had higher levels of externalizing problems. On average, children experienced a linear decrease and slight quadratic increase in externalizing problems across age. There was a greater rate of linear decrease for Indian, Pakistani, and Bangladeshi children than for white children.

**Table 4:** Growth curve model for externalizing problems

Parameter	Coefficient	Standard error
Model 5		
Intercept	10.42***	0.13
Age (linear slope)	−0.88***	0.014
Age squared (quadratic slope)	0.042***	0.0008
Indian	0.14	0.20
Pakistani	0.46**^a^	0.17
Bangladeshi	0.27	0.30
Black Caribbean	0.43*^a^	0.20
Black African	−0.43	0.24
Other/Mixed	0.16	0.22
Child sex	−0.98***	0.047
Household income	−0.37***	0.020
Parental education	−0.37***	0.020
Maternal mental health	0.39***	0.014
Maternal immigrant status	0.25**	0.097
Indian × Age	−0.048*^a^	0.019
Pakistani × Age	−0.085***^a^	0.016
Bangladeshi × Age	−0.091***^a^	0.027
Black Caribbean × Age	−0.0074	0.020
Black African × Age	−0.027	0.024
Other/mixed × Age	−0.026	0.021

*p < 0.05, **p < 0.01, ***p < 0.001

^a^Significant difference between respective group and white group

**Fig. 2:**
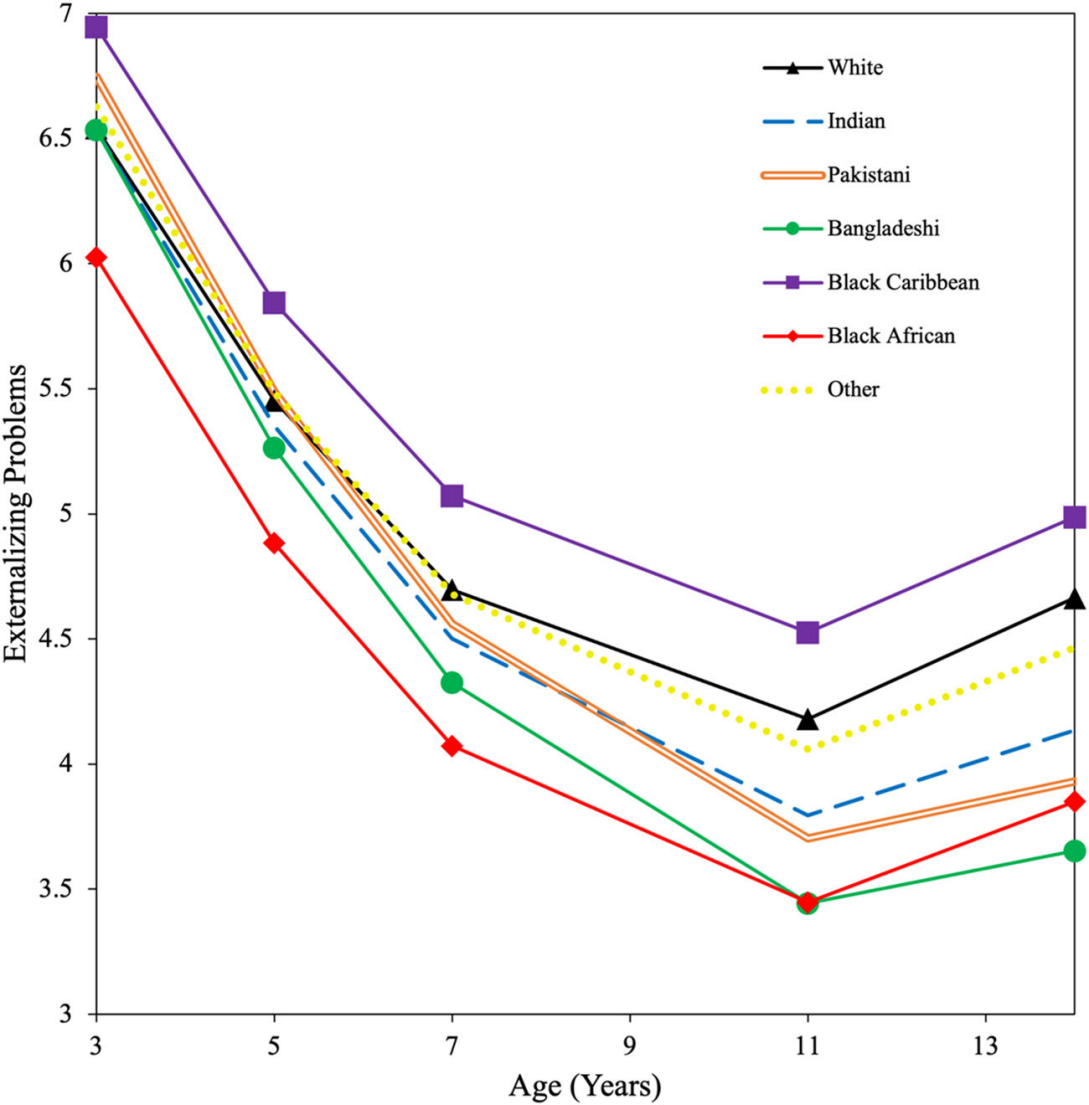
Growth curves for externalizing problems by ethnicity

Figure 2 shows the trajectories of externalizing problems according to ethnicity. From ages 3 to 14, Black Caribbean children showed higher, other/mixed children showed similar, and Black African children showed lower, levels of externalizing problems than white children. Pakistani children showed higher levels than white children at age 3, similar levels from ages 5 to 7, and lower levels from ages 11 to 14. Indian and Bangladeshi children showed similar levels than white children from ages 3 to 7, and lower levels from ages 11 to 14. All children showed an increase in externalizing problems from age 11. By age 14, Black Caribbean adolescents showed the highest problem levels of all groups, while Bangladeshi adolescents showed the lowest.

## Discussion

Existing evidence shows that there are developmental differences in mental health problems. However, less is known about the development of mental health problems in ethnic minority children, particularly at the population level. Using data from UK Millennium Cohort Study, a nationally representative and ethnically diverse survey, this study examined ethnic differences in children’s (a) cross-sectional mental health problems and (b) trajectories of mental health from ages 3 to 14 years. Significant differences were found in internalizing and externalizing problems between ethnic minority and white children, signaling ethnic inequalities in child mental health, particularly in early childhood. Growth curve modeling revealed that ethnic minority children followed different developmental trajectories of internalizing and externalizing problems than white children, either in terms of the mean-level and/or rate of change across age. These differences were not explained by child sex, socioeconomic status, maternal depressive symptoms, and maternal immigrant status, highlighting the need for further research exploring the factors that underpin ethnic differences in child mental health.

### Ethnic Differences in Mental Health Problems

Ethnic minority, except Indian, children had more internalizing problems than white children. This difference has been identified in previous studies examining children aged 3 (Platt, 2012) and 7 (Zilanawala et al., 2015). Although the findings were mostly in line with prior research, there were some notable discrepancies. First, this study found mixed ethnic differences in internalizing problems in early childhood, while Platt (2012) found no difference in mental health scores between mixed ethnicity and white 3-year-olds. This inconsistency in the literature is likely to reflect methodological differences, such as different configurations of the ‘mixed’ ethnic group. Unlike Platt (2012), this paper does not use a detailed classification of mixed ethnicity, making it difficult to directly compare the findings. Second, this study found an internalizing disadvantage for Black African children in early childhood, while Platt (2012) found that Black African children had fewer composite mental health problems. This difference may arise from the fact that this study disaggregates mental health scores into internalizing and externalizing scores, increasing the ability to detect previously masked ethnic differences.

By using a longer age range and taking a developmental perspective, this study demonstrates that the internalizing disadvantage noted at ages 3 and 7 (Platt, 2012; Zilanawala et al., 2015) persists into adolescence for children of Pakistani and Bangladeshi origin, but not for those of Black Caribbean, Black African, or other/mixed ethnicity origin. One possible explanation for this ethnic difference is that there may be factors related to the family or schooling environment which influence resilience, personal experiences, and coping strategies (Patalay, 2015), contributing differently to symptom maintenance. Recent reports show that young Muslims in the UK, with particular emphasis on those from the South Asian community, face several challenges as they emerge into adolescence including Islamophobia, social exclusion, negative stereotypes, cultural barriers, and identity issues (Stevenson et al., 2017). These barriers to positive schooling experiences for Muslims in the UK, which may have become exacerbated in recent years due to the rise in religiously motivated hate crimes (ONS, 2020a) and anti-Muslim sentiment (Dadabhoy, 2018), could explain the disproportionate risk for internalizing problems in British Pakistani and Bangladeshi adolescents, given that 67% of the UK Muslim population are from South Asian backgrounds (ONS, 2011c).

Pakistani and Black Caribbean children also had more externalizing problems than white children; corroborating findings from previous research examining children aged 7 (Zilanawala et al., 2015). This study adds to the literature by demonstrating that the externalizing disadvantage persists into adolescence for Black Caribbean children, but not for Pakistani children. For Pakistani children, there seems to be a shift in pre-adolescence from having a co-occurring disadvantage (i.e., higher levels of both internalizing and externalizing problems) to having a pure-internalizing disadvantage (i.e., higher levels of internalizing problems only, in comparison to white children). The reason for this shift is not clear, and future studies should aim to illuminate the factors that underlie this difference. Below is a discussion of the growth curve trajectories, which were estimated in order to determine whether there are ethnic differences when potentially confounding factors (i.e., child sex, socioeconomic status, maternal depressive symptoms, and maternal immigrant status) are accounted for.

### Ethnic Differences in Developmental Trajectories of Internalizing Problems

Although developmental patterns of internalizing problems were generally similar across ethnic groups (i.e., decreased from early to late childhood and then increased thereafter), differences were observed in both mean levels of symptoms and rates of change. Previous longitudinal studies on ethnic differences in child mental health have generally not used detailed ethnic categories. The findings from the current study indicate that Indian, Pakistani, Bangladeshi, Black Caribbean, Black African, and other/mixed children aged 3 (disaggregated groups) have higher levels of internalizing problems than white children, signaling poorer mental health outcomes for ethnic minorities. This disadvantage persisted until age 7 for most minority groups, and by age 14, Black Caribbean adolescents continued to show higher levels of problems, contrary to the cross-sectional findings. Research has documented an internalizing disadvantage for Pakistani, Bangladeshi, and Black Caribbean children aged 7, which remained after adjusting for multiple markers of SES (Zilanawala et al., 2015). Taken together, the findings from the current study and prior work highlight that ethnic minority children are at greater risk of internalizing problems than their white counterparts. This difference is not explained by child sex, SES, maternal mental health, and maternal immigrant status, suggesting that there may be other factors linked to minority status which influence socioemotional behavior. Although additional research is needed to ascertain specific processes, risk factors for internalizing problems in ethnic minority children could be explained by the critical race theory which explores the unequal distribution of resources, diagnosis and treatment of mental health conditions, and societal and political manifestations as the result of racial stratification (Moodley et al., 2017).

In line with previous longitudinal research (Costello et al., 2011), this study also found that children, on average, followed a decreasing trajectory of internalizing problems from ages 3 to 14, with symptoms increasing slightly as they entered adolescence. This study is the first to demonstrate, using a nationally representative sample, that there are developmental differences in the rate of decrease, according to ethnicity. Ethnic minority children, with the exception of Black Caribbeans, experienced a greater rate of linear decrease across age than white children. While white children showed an increase in internalizing problems from age 7, ethnic minority (except Black Caribbean and other/mixed) children showed an increase from age 11. The reason for this ethnic difference has not been elucidated, although the increase in all groups is likely to coincide with the onset of puberty and associated academic transitions (Sawyer et al., 2012).

### Ethnic Differences in Developmental Trajectories of Externalizing Problems

Likewise, developmental patterns of externalizing problems were similar across ethnic groups (i.e., decreased from early to late childhood and then increased thereafter), but differences were observed in both mean levels of symptoms and rates of change. At age 3, Pakistani and Black Caribbean children showed higher levels of externalizing problems than white children. This difference is only evident at age 3 for Pakistani children but seems to remain higher for Black Caribbean children from ages 3 to 14, which is of concern. Although the reason for this ethnic difference has not been elucidated, research has highlighted the role of school and institutional racism in explaining higher levels of conduct problems in Black Caribbean children in Canada (Rousseau et al., 2008). In the UK context, Black Caribbean children face a number of structural and social inequalities including stereotyping and microaggressions (Doharty, 2019), institutional racism in schools (Gillborn, 2008), low teacher expectations (Strand, 2012), disproportionate school exclusions (ONS, 2021), disproportionate police stop and search (ONS, 2020b), and negative media profiling (Malik, 2001). These factors have been shown to perpetuate the Black Caribbean attainment gap in the UK (Demie & McLean, 2017); thus, they could play some role in explaining their disproportionate risk for externalizing problems.

Another explanation considers migration history and transgenerational consequences of racial discrimination and social marginalization (Schoon & Melis, 2019). It is well known that Black Caribbeans were the first non-white group to settle in post-war Britain in the 1950s, with many migrants citing experiences of hostility, resentment, and overt racism on arrival (Whitfield, 2006), as well as difficulties with low paid, low status work, poor housing, and institutional racism in the British police and educational institutions (Arnold, 2011). In accordance with theories of cumulative disadvantage (Diprete & Eirich, 2006), several authors have postulated that systemic failure and unresolved historical trauma can inflict significant psychosocial burden on successive generations (Davis, 2007; Sullivan, 2013) through direct (i.e., changes in family structure; economic dependence) and indirect (i.e., effects on identity formation) influences (Nicolas & Wheatley, 2013). Enhanced understanding of the intergenerational effects of trauma among second and third generation Black Caribbean immigrants may yield insights into novel ways to promote child mental health equity in this group.

In line with previous longitudinal research (Pingault et al., 2015; Miner & Clarke-Stewart, 2008), this study also found that children, on average, followed a decreasing trajectory of externalizing problems from ages 3 to 14, with symptoms increasing slightly as they entered adolescence. This study is the first in the UK to demonstrate that there are developmental differences in the rate of decrease, according to ethnicity. Indian, Pakistani, and Bangladeshi children experienced a greater rate of linear decrease across age than white children. The slight increase in externalizing problems at age 11, which did not differ according to ethnicity, is likely to coincide with the onset of puberty and transition to secondary education (Sawyer et al., 2012).

### Implications

The findings from this study suggest that the underrepresentation of Black Caribbean children with emotional and behavioral disorders within voluntary UK mental health services (Edbrooke-Childs et al., 2016) could reflect unmet need, rather than lower prevalence. In the UK, mental health professionals are predominately of white British background (Health and Social Care Information Centre, 2013), with many citing a lack of confidence and inability to detect symptoms of anxiety and depression in individuals of African ancestral origin (Fernando & Keating, 2008). By showing that Black Caribbeans are at greater risk of developing both internalizing and externalizing problems from early childhood to mid adolescence, this study illuminates the need for practitioners of Caribbean descent and stresses the importance of addressing institutional barriers that prevent Black applicants from pursuing clinical psychology (i.e., issues relating to social capital, finance, and the selection process; Tong et al., 2019). The current mental health system in the UK has few Black clinical psychologists, and thus, many lack ‘insider knowledge’ relating to Black Caribbean culture (Edge, 2010), negatively affecting both quality of care (Mclean et al., 2003) and treatment outcomes (de Haan et al., 2018). It is also well established that the Black community in the UK are less likely to engage with voluntary mental health services due to experiences of stigma and gaslighting (Keating et al., 2002), as well as concerns regarding the lack of cultural awareness (Memon et al., 2016). Ethnic differences observed in the current study highlight the importance of adequate cultural competence training (i.e., efforts to increase the racial literacy of staff) and diversity in the division of clinical psychology. Diversity of the clinical psychology workforce is an important contributing factor in ensuring that children and adolescents are able to access voluntary mental health services that reflect their own particular culture and personal identities.

### Limitations and Future Research

This study is not without limitation. First, this study relied on parent-reported mental health data, with a lower than acceptable internal reliability for internalizing problems at age 3. As parent-reported data may be subject to informant bias, the ethnic differences that emerged may partially reflect adults’ perspectives on child behaviors, which is heavily influenced by culture and social customs. Although it can be argued that teachers have a more limited familiarity with a child’s development than parents, as well as an implicit tendency to make harsher assessments of black than white students (Mortenson, 2018), the MCS collected teacher reported SDQ scores at ages 11 and 14 and future research should use multi-informant designs to validate the current findings. Second, this study controlled for only two markers of socioeconomic status (SES). Although research suggests that equivalized income has the strongest impact on children’s mental health problems (Zilanawala et al., 2015), future research should explore the role of other markers of SES, such as the Index of Multiple Deprivation (IMD) and parents’ employment status. Third, although the analytic sample was large and nationally representative, the sample sizes of the ethnic minority groups were significantly smaller than that of the white group, restricting the ability to draw conclusions from the analyses due to reduced statistical power. Similarly, this study collapsed ‘mixed’ ethnicity and ‘other’ ethnicity (i.e., Arab, Chinese, Sri Lankan) children into a single, broad group, making it difficult to permit meaningful analysis. Fourth, attrition rates were significantly higher for ethnic minority children than for white children. As the predictors of attrition are similar to the predictors of child psychopathology, it is possible that the proportion of ethnic minority children with severe symptoms is underestimated. Selective attrition was, however, mitigated with the use of the MCS survey team’s attrition weights, as well as the use of growth curve models, which estimate trajectories for cohort members despite missing data. Fifth, although this study captures developmental trajectories across five data points, the MCS does not provide data for cohort members at age 9. As more distinct differences across ethnic groups tend to emerge from pre-adolescence to adolescence, missing age 9 is potentially omitting important transient changes across groups. Sixth, this study was not able to explore possible racial explanations for observed ethnic inequalities. In the UK, significantly higher rates of psychiatric disorder are observed in ethnic minority adults than white adults (Department of Health, 2011). Systemic racism and experiences of discrimination are overwhelmingly cited as major and consistent barriers to mental wellbeing in ethnic minority adults (Arday, 2018; Wallace et al., 2016). Whether racial discrimination plays a role in ethnic minority children’s mental health remains unknown. The MCS collects a proxy measure of perceived racism and future research should adjust for this to see if it explains any ethnic differences. Qualitative interviews would also be useful in this context. Finally, the data for MCS7 (where children are aged 17) were not available on the public domain at the time of this study. This means that mental health was not explored in late adolescence or early adulthood, a time at which ethnic minority disadvantages may reappear or become exacerbated by prolonged exposure to adversity and discrimination in places of study and work (Benner et al., 2018; Wallace et al., 2016). Future longitudinal research should assess mental health trajectories through adolescence into early adulthood to see if there are notable changes.

## Conclusion

There is a dearth of research examining the developmental course of internalizing and externalizing problems in ethnic minority children in the UK from early childhood to adolescence. Using a nationally representative sample and detailed ethnic classification, this study filled this gap, revealing that ethnic minority children followed significantly different developmental trajectories compared with their white counterparts. The findings showed that all ethnic minority groups were disadvantaged in terms of their internalizing problems, and Pakistanis and Black Caribbeans in terms of their externalizing problems. Given the lower rate of decrease for white children, however, white children had similar or higher levels of mental health problems by age 14. One note of concern is Black Caribbean adolescents, who seemed to have the highest levels of internalizing and externalizing problems compared to all groups at age 14. Given that this group is often subsumed in a larger and more diverse ethnic minority group which includes Black Africans in the UK, more research studies examining risk factors of mental health problems for Black Caribbean adolescents are needed. Overall, this exploration contributes to a nuanced understanding of the longitudinal course of mental health in ethnic minority children in the UK through the identification of ethnic inequalities in child psychopathology, as well as distinct trajectories of mental health among ethnic minority groups from early childhood to mid adolescence. Studying these heterogenous pathways is of critical importance, given that such explorations can improve our insight into the etiology and development of mental health problems in ethnic minority children, inform culturally competent diagnostic and screening procedures, and help tailor evidence-based clinical interventions for ethnic minority populations in the UK.
